# Technology adoption performance evaluation applied to testing industrial REST APIs

**DOI:** 10.1007/s10515-024-00477-2

**Published:** 2024-12-02

**Authors:** Alexander Poth, Olsi Rrjolli, Andrea Arcuri

**Affiliations:** 1 Volkswagen AG, Wolfsburg, Germany; 2 Kristiania and OsloMet, Oslo, Norway

**Keywords:** Quality engineering, Requirements-based testing, Test automation, Test-case generation, Benchmarking, API validation, SBST, LLM

## Abstract

Testing is an important task within software development. To write test cases and integrate them into an automated test suite requires a significant amount of work. Given a set of requirements and specifications of a software, testing is needed to verify its correctness. When done manually, it is an expensive and error prone task. To facilitate such work, automated test-case generation via tools could be useful. Test-case generation can be facilitated by deterministic algorithm-driven approaches or non-deterministic approaches such as with AI (e.g., evolutionary and LLM). The different approaches come with their strengths and weaknesses, which must be considered when integrating these approaches into a product test procedure in industry. Several novel testing techniques and tools have been developed in academia and industry, but how effective they are and how to integrate them in real-world large industrial scenarios is still unclear. In this paper, a systematic approach is presented to evaluate test-case generation methodologies and integrate them into a scalable enterprise setup. The specific context is black-box testing of REST APIs, based on their OpenAPI schemas. The aim is to facilitate IT product development and service delivery. The proposed Technology Adoption Performance Evaluation (TAPE) approach is evaluated by a case study within the Group IT of Volkswagen AG. We evaluated existing tools such as OpenAPI Generator, EvoMaster and StarCoder which are built on different technologies. Our results show that these tools are of benefit for test engineers to facilitate test-case specification and design within the Group IT of Volkswagen AG.

## Introduction

In the software development process testing is an established required task. In complex software products testing becomes a challenging task (Garousi et al. 2020), which demands a significant amount of time and resources. Especially with focus on the life-cycle of the software with its releases, testing becomes a repetitive task at each new release. To reduce time in the release process, test automation is widely established to verify if the requirements of the software are satisfied. However, usually such automation is limited only to the test execution (e.g., via test scripts or test suites written with libraries such as JUnit, for Java). Still, these test scripts need to be written and maintained manually by test engineers. To reduce the time and effort during the test-case specification, design and implementation, a test-case generator could facilitate the work of the test engineers.

In the literature, there exists a vast number of options to start a facilitation journey for introducing test-case generation in real-world industrial contexts. From an organizational perspective, the introduction of test-case generation is an innovation. Innovation is defined by Baregheh et al. (2009) with: “*Innovation is the multi-stage process whereby organizations transform ideas into new/improved products, service or processes, in order to advance, compete and differentiate themselves successfully in their marketplace*”. In most cases in practice, a focus is set, i.e., not everything at the same time, and a value driven approach, by considering feasibility constraints, is selected to target the marketplace. To measure progress, and plan the next steps of this innovation journey, a systematic approach is needed. As test-case generation can be realized with different technologies, the approach must be technology independent. Furthermore, the approach should be generic for usage also in other scenarios beyond test-case generation or testing.

To enhance service development and improve the process of delivering new releases, at Volkswagen AG the Technology Adoption Performance Evaluation (TAPE) approach was developed and evaluated as a generic approach, which we introduce in this paper. The goal is to simplify the evaluation and integration of novel techniques (e.g., the use of test case generators) into industrial practice at Volkswagen Group IT. As TAPE facilitates technology management, it also needs an open architecture and design to evaluate extended technologies or technology-based products, e.g., services with incremental releases. Also, TAPE is open about the specific goals of the technology adoption, e.g., facilitate users or optimize workflow costs. So, TAPE helps to identify technologies that fit to a specific use case. It is a lean alternative to complex technology assessments and it can be adopted to the specific usage context.

The specific context analyzed in this paper is the black-box testing of REST APIs as part of the IT system testing. System testing is defined as “*Testing an integrated system to verify that it meets specified requirements*” (ISTQB glossary 2024). Test engineers receive the OpenAPI (OpenAPI 2024) schemas of the APIs implemented by different development teams at Volkswagen AG, and then need to write automated tests to verify those requirements at each new API release. Black-box testing is defined as “*A procedure to derive and/or select test cases based on an analysis of the specification, either functional or non-functional, of a component or system without reference to its internal structure*” (ISTQB glossary 2024). The engineers have to derive test requirements, identify acceptance criteria from the specification for acceptance tests and validate with the test suites the requirement specifications. As this is a very expensive task, companies are eager to evaluate and introduce novel techniques that can reduce cost and improve quality, like test case generators. In particular, in this paper we evaluated three different approaches: deterministic (using OpenAPI Generator (Generator 2023)), evolutionary (using EvoMaster (EvoMaster 2024)) and with large-language-models (LLM) (using StarCoder (StarCoder 2024) for evaluation). Two REST APIs developed at Volkswagen AG were used as an initial case study. These techniques are compared with the existing test cases manually written for these APIs, to assess their strengths and limitations.

This work makes the following major contributions:

A) Novel methodology
B) We propose an approach to evaluate the performance of technology adoption in the context of test-case specification and automation facilitation with the TAPE evaluation approach.
C) We model generic building blocks for TAPE to apply the approach within different scenarios and environments.
D) Study for the test-case generation use case
E) We adopt different technologies in the context of test-case generation for system tests. We introduce an architecture and design (called Tanius) fostering incremental extensions of the service to keep a high delivery performance.
F) We conducted an empirical study for system test case generation for requirements verification within a large enterprise IT setup.
G) To the best of our knowledge, this is the first work in the literature (Golmohammadi et al. 2022) where REST API fuzzers (tools that send inputs to a system and monitor the system behavior) are compared in industry, by test engineers, with existing manually written tests by those same engineers.
H) The article begins by introducing the TAPE evaluation approach, which demonstrates how various solutions can be assessed. The TAPE approach is designed as a generic benchmarking method capable of evaluating different technologies using a domain-specific benchmark suite, targeting relevant aspects and scenarios for a given business domain. In the case study, a practical application within the context of Testing as a Service (TaaS) in the IT testing domain is presented. TaaS serves as a tool for IT system testing, where different solution approaches based on specific technologies can be integrated. To incorporate the most suitable solution approaches, several options for test-case generation for REST-API testing are evaluated using TAPE. This evaluation aims to integrate the most promising test-generation solution into TaaS, thereby maximizing its benefits in future IT testing projects. The evaluation with TAPE to select solutions highlights the practical value of domain-specific benchmarking, ensuring that TaaS incorporates the most appropriate solutions. The discussion provides insights gained from benchmarking different test-case generators using TAPE.

## Background and motivation

With new technologies such as Cloud Computing and Artificial Intelligence (AI), organizations have to find ways to adopt these technologies to benefit from the chances offered to improve their products and services. However, technologies such as AI are complex and not easy to manage. The complexity comes in this case from the large number of specific technologies and approaches which are part of AI to build, run and maintain the technology. For example, an Evolutionary Algorithm based approach is very different from the use of Large Language Models. An organization has to decide early which parts of the technology should be “only” used, and which parts should be actively designed and tailored for their specific needs. In case of AI, the model and data centric approach can be distinguished (Hamid 2022). For example, a small organization unit can have expertise and knowledge to manage domain specific data. So, the organization focuses on a data centric approach by improving data quality and quantity over time. On the other hand, a small organization unit would not be able or willing to build expertise in modeling Machine Learning (ML) networks or in algorithm engineering. However, basic knowledge about models has to be built to understand their challenges and limitations, which are also affecting the data centric approach.

Within this setup, it is important to have an approach guiding enterprises through the technology adoption. It is difficult to define a-priori a set of (static) requirements for a specification document. There is a high risk that requirements become suddenly outdated with the speed of the evolution of the technologies. Or the requirements are not specific and addressing only generic objectives. The proposed approach has to be open to different adoption scenarios of the technology. However, it has to be specific enough to be able to measure the performance of a specific scenario adoption.

With the TAPE approach and the example scenario “test-case generation” implemented with Tanius, presented in this paper, a two-year journey at the Group IT of Volkswagen AG is reflected in this work.

## TAPE: approach and methodology

To better understand how to manage the technology adoption journey, first the TAPE approach is developed. This development was realized by a design process which results in (1) the generic model, (2) the practical model, and (3) the scenario instantiated model for test-case generation, as discussed in more details in the next subsections. The three design process result presentations are structured by the task which describes the objective, the assumptions for the task context and result specific aspects. The scenario instantiated model was used for evaluation, in particular with a case study from the Volkswagen Group IT. This evaluation shows the performance of the approach. It is the base used to discuss the benefits and limitations of the proposed technology adoption approach.

### Generic model

One of the main goals of requirements engineering is to ensure that the stakeholders’ needs are addressed. The objective of the generic model is to optimize a given process with automation via IT. The relevant aspects for optimization are depending on the use case which is evaluated and benchmarked.

#### Task

Optimize a given process with automation via IT. As an existing process, it defines a status-quo. The status-quo can be measured and used as baseline (ground truth). Core measures are the costs ($) and the time (t) needed to perform the process, i.e., process steps. Example for a task is the following practical scenario: facilitate the test-case generation for API system tests based on OpenAPI specifications.

#### Assumptions and core-model

The generic model is by design open to handle scenarios in which performance benefits are expected by automation via IT. To support business processes with IT, it is useful to culminate with the technology evaluation and benchmarking in some business metrics such as costs. Two aspects are relevant in a cost value analysis—the development (of a release) and operating phase. To handle these topics, an ideal environment model is developed. The model is based on some assumptions, as discussed next.

##### Releases

A release contains all development phases of a product increment, which results in an executable service. A release is realized by a number of resources (e.g., #developers) within a specific timeframe. This can be release specific, or always the same in the case that a stable team delivers in a fixed release plan, such as Sprints in Scrum. Each release has its cost computed by multiplication of the resources with their cost and with the time-frame. In this context, a release is defined by its fix-costs (ReleaseCosts) to deliver a set of value units (ValueUnit). A ValueUnit is the outcome of the process which is optimized. The ValueUnit benefit is the result of the user value after direct associated unit runtime, respectively execution costs are subtracted. In the case of test-automation, the generated test-cases are the user value units. To have a clear expectation of what is a “good” test-case or test-suite, a baseline such as a “ground truth” ValueUnit is useful.

##### Operation

The generic model assumes an ideal workload during the operation of a release. Ideal workloads have no static footprint. An ideal workload commissions the needed resources for the requested value units on-demand (ResourceDemand). ResourceDemand can be measured in process relevant resources such as CPU and RAM allocation and their assigned costs—e.g., these resources fit fine to the pay-as-you-go approach of cloud providers. This assumes an ideal elastic scaling. In this context, operation is defined by the variable costs per consumed workload value unit.

The benefit of this generic model is as simple as these calculations:

1 $${\text{AddedValue}} = {\text{ValueUnit}}{-}{\text{ResourceDemand}}$$

2 $${\text{CostOfValueUnit}} = \# {\text{AddedValue}}/{\text{ReleaseCosts}}$$

(by counting AddedValue units for a useful timeframe, e.g., by counting the API requests for a ValueUnit over a release deployment in the case of an established cadence such as Sprints.)

It is possible to extend this simple model with, for example, operation fix costs for the Ops-team, core-services not directly depending on workload, etc. Also, the release fix-costs can be discounted, respectively deprecated, over larger timeframes than the time to the next release. However, as long the complexity is not needed it should be avoided.

##### Value units

The specific part of the model is to identify the value units for the given scenario. This is a “creative task” which has to be done for each scenario. The objective is to identify value units addressing the following aspects oriented on (Kistowski et al. 2015):

- Relevance: the value unit is relevant in the business context.
- Reproducibility: the value unit measures can be reproduced.
- Fairness: does not limit other “ways of work”.
- Verifiability: the value unit can be easily verified.
- Usability: the value unit measure does not need experts.

Especially, fairness is important in the focused context, because with new technologies the “way of work” can differ significantly for the established workflow and its activities. An example for a ValueUnit could be the provisioning of a specific test-execution environment for test-suite execution. The ValueUnit of the example is defined by the configuration options and explicit countable by the user requests.

As not everything can be automated with the same effort, it is useful to start in promising areas. Guiding questions to reach fast positive effects are: what gives high value? What has potentially low operation effects? What has potentially low development efforts? The first two questions are scaling with the value units and so it is important to focus on them first. The development is a fixed cost, which becomes more important in case of small scaling numbers.

**Fig. 1:**
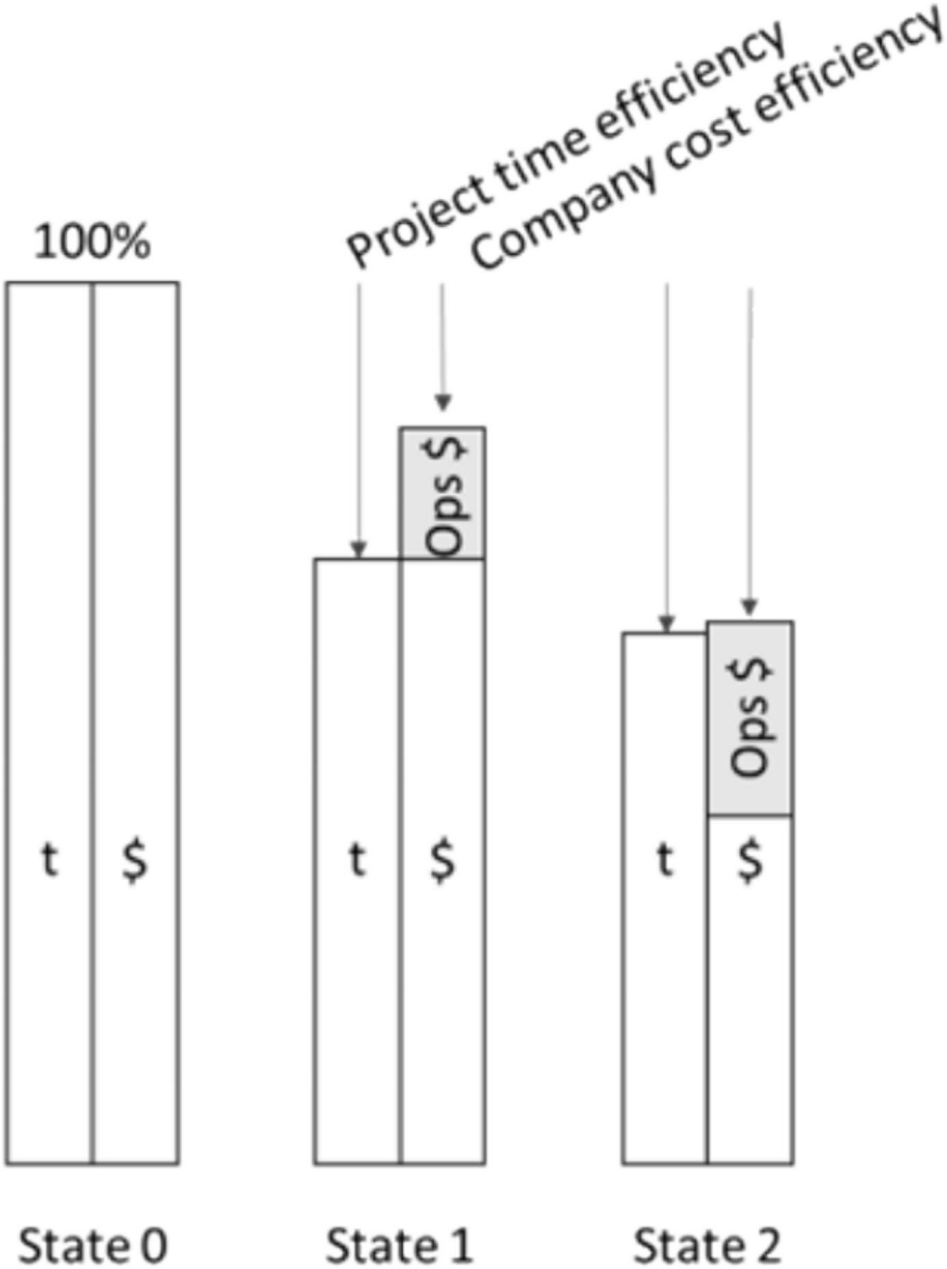
Immprovement of the time- and cost-performance over states (100% baseline and two release) for a value unit

Figure 1 presents an example with a State 0 which uses the un-improved process as initial baseline and two automation releases (State 1 and 2). The time (t) pillars of the releases show the saved time per value unit from the user, respectively project perspective. The cost ($) pillar distinguishes between project costs and service costs. The project costs for the task performing person do not have to be in the same relation as the time savings, in the case that, for example, a less qualified and less expensive person can perform the task with the service facilitation. The ops costs are the resources allocated for performing the workload per value unit (delivery costs). They depend on the used technology and implementation. For example, in State 2 the relative change between the ops and project cost can come from using an advanced technology such as a LLM to facilitate test-case generation. So, a junior test engineer could perform the task instead of a senior test-engineer in State 1 by an overall time optimization between the two releases. For example, IT experts are expensive and hard to hire. Having more ops costs (e.g., for TPU hardware infrastructure) would not be a problem if it can free valuable time from expensive, senior experts. In these cases, it can be considered a significant automation improvement. In the State 1, the same qualified person is still needed to perform the task, so the time optimization correlates with the project cost optimization and on-top the automation ops cost are added. A tipping point for future states is reached when the ops costs are growing more than the projects savings—this case only works in real life when the time saving legitimates the additional overall costs.

We use the term “State” instead of “Release” here, for two main reasons. First, between the adoption of a tool and its update in two different states, such a tool might have gone through several of its own releases. In other words, we do not necessarily update a tool every single time it has a new release. Second, as the baseline is often a manual reference without automation, the word “release” here might be confusing.

Practitioner note: This presented generic model fits fine, e.g., for event driven serverless architectures operated by a cloud provider. Only a value unit invocation (e.g., generate a test-code stub based on a given OpenAPI specification) initiates resource allocations for handling the workload request with the ops costs. For other architectures, it might be that fix costs for ops have to be considered if they are significant.

#### Practical model

Requirements have to be specific for the context and measurable. The practical model refines the generic model to be applicable in a specific context.

##### Task

Extend the generic model by defining a more practical evaluation approach. In the real-world, a scenario’s performance depends not only on one value unit with one attribute measured in one case. Often, for a scenario there exist less and more complex value units, attributes and cases and some are performed more often than others, etc.

##### Assumptions and benchmark-model

The generic approach is extended by adding to the value units the relevant attributes, which are distinguishing the real context. The assumption is that attributes could be size, complexity, etc. For each attribute, relevant cases are identified such as slow or fast, easy or difficult. However, to avoid unnecessary complexity, the number of cases per value unit, e.g., attribute, should be as small as possible. A practicable amount for cases is the T-shirt sizing (Mallidi and Sharma 2021), with for example S, M, L and XL. Each size represents a relevant value option of the attribute. An example could be S < 10 s, M < 20 s, L < 30 s and XL everything which takes longer to perform.

Objective is to focus on core attributes of a value unit and a minimized set of cases per attribute to ensure efficiency by using the model in practice.

##### Benchmark suite

Grouping the cases together defines a value unit specific benchmark suite. As the performance of the benchmark suite depends on humans performing tasks, a variation can be assumed. Therefore, it is recommended to perform the benchmark suite at least a few times to see what the spread is. Depending on the determinism of the automation, this repetition to measure the variation can be useful, too. However, in practice, nobody will do a service request more than a few times to get an acceptable result. Ideally, it is useful to repeat the benchmark suite often to get average and median values. In practice, in most cases this is too expensive.

**Fig. 2:**
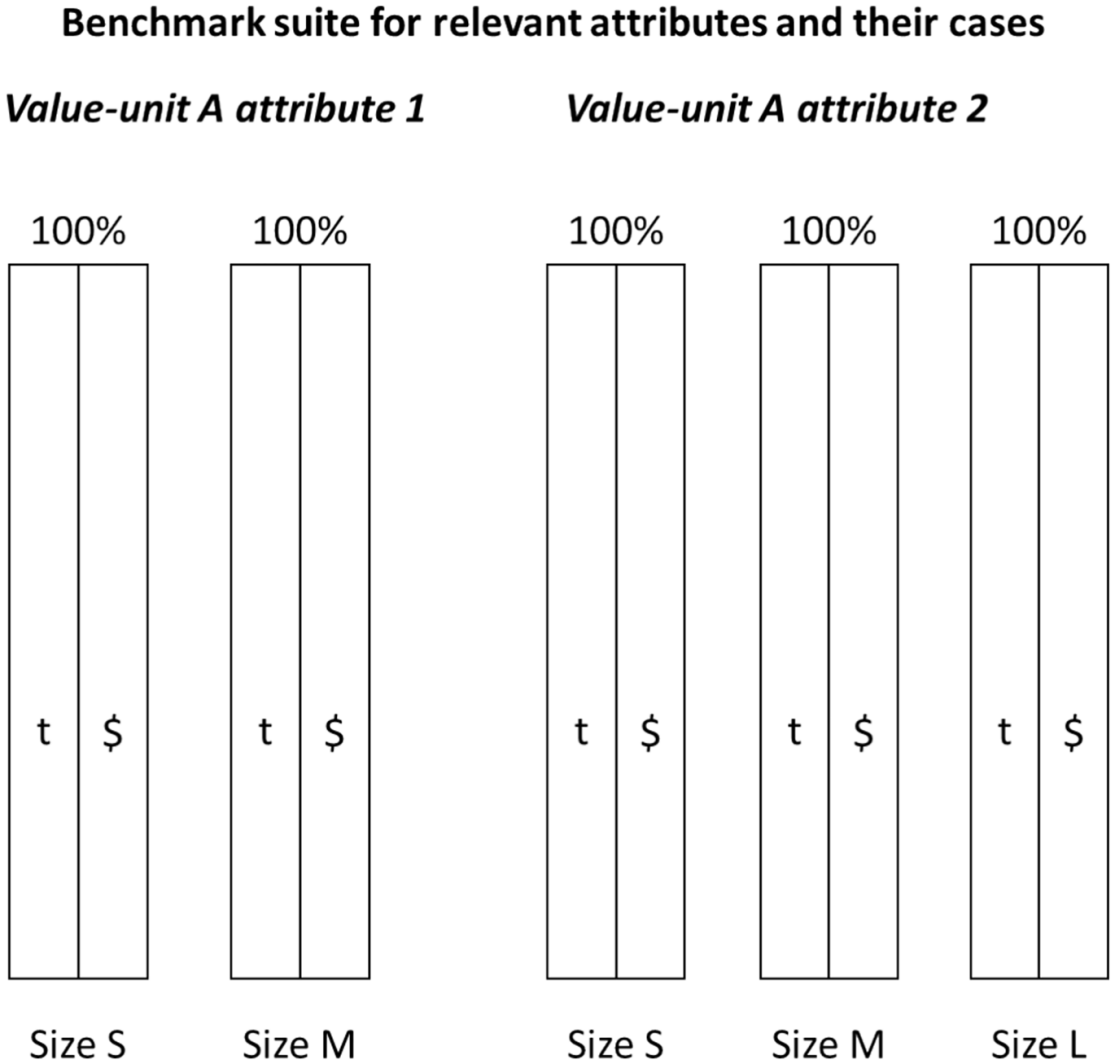
Example benchmark suite for 2 attributes and their cases for a value unit as baselines

Figure 2 shows that the benchmarking conduction effort grows significantly for different attributes and their cases in comparison to the simpler generic model. Attribute 1 could be for example without (size S) and with (size M) data parameters and attribute 2 complexity with a simple (size S), normal (size M) and complex (size L) case. For the case that measurement variation is possible, and there is a need to identify the spread, the effort grows more.

Practitioner note: the usage of a relative scale, e.g., a percentage scale which defines 100% as baseline, makes general communication more simple. This is motivated because, in large companies, often absolute performance numbers are not shared and commonly understood broadly. As presented in Fig. 2, all attributes and cases have in the State 0 a 100% value per definition, as it is the baseline. The benchmarking of State 1 etc. should show improvements to this baseline.

#### Scenario instantiated model

To be able to validate the refined practical model to a specific context, the instantiated model defines the specific requirements which are “observed”.

##### Task

Extend the practical model to an applicable model for a specific scenario. The example scenario is test-case generation.

##### Assumptions

The workflow with test-case generation facilitation contains at least the following tasks, which are different or added to a complete traditional (manual) test case design and implementation:

- The test-case generator has to be “fed” with inputs by a test engineer.
- The output from the test-case generator has to be understood and validated by the test-engineer.
- The test-engineer has to fill the gap between the test-generators output and the needed quality of the test-cases. This effort contains “bug-fixing” (not all generated output might be correct or usable) and adding missing parts of the test-cases.

**Fig. 3:**
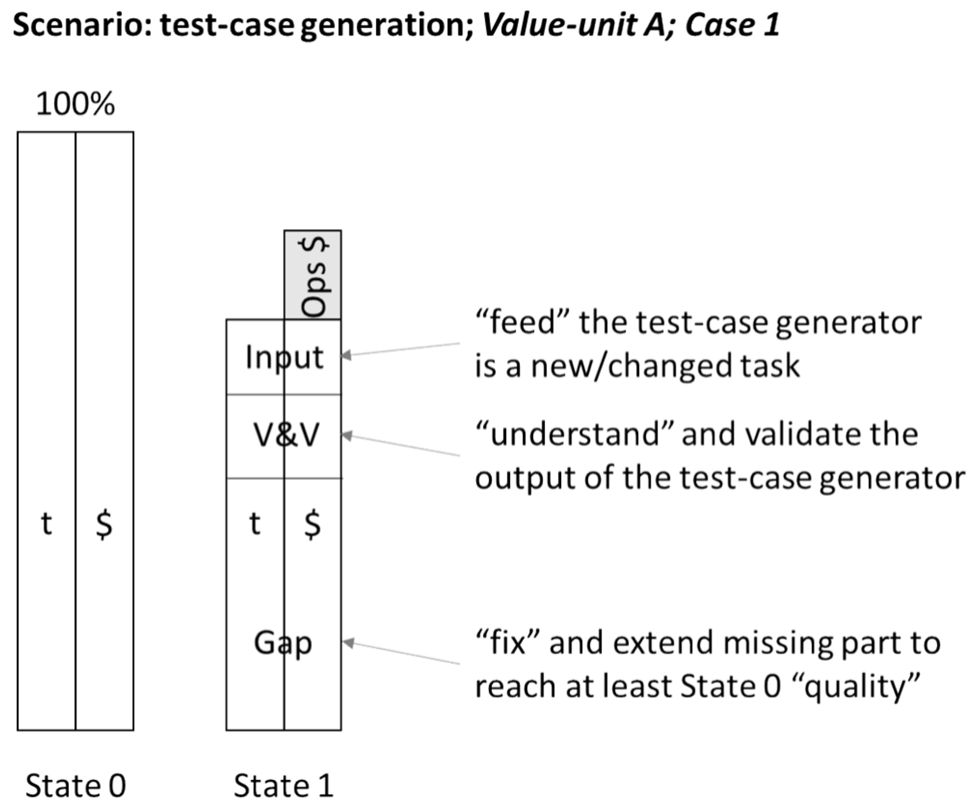
Example of a value unit and its parts respectively tasks: input, V&V and gap

In Fig. 3 the three tasks are called *input*, *V&V* and *gap*. The figure presents them as parts of State 1.

##### Benchmark suite

The benchmark suite has at least to address cases for different complex test-cases. Complexity can come from complex business logic, which has to be handled in the test-cases. Also, a major source of complexity in testing is the test data. The tests have to be able to handle different test data sets to stimulate the System Under Test (SUT), for example with a positive and a negative test run.

**Fig. 4:**
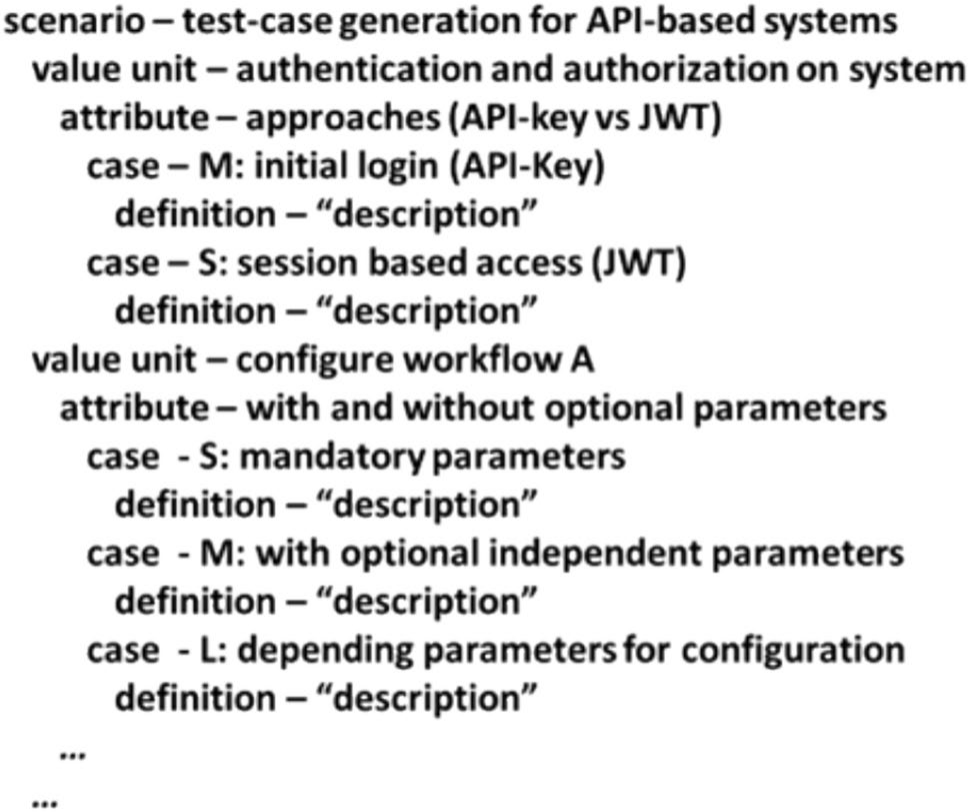
Example of a refined TAPE scenario with value units, attributes and cases—the cases building the benchmark suite

The refinement of the benchmark has to focus on the scenario which is in scope and handle at least the attribute complexity with adequate cases. Figure 4 presents an example for the system test-case generation scenario for APIs. The example with the complexity M for the initial login presents that this is more than the session based access which is sized as S. The value unit example with workflows is more abstract but shows that the interaction with APIs can become complex if additional parameters are needed and if the parameters have dependencies it becomes an additional complexity level (which often needs “insider knowledge” about what correlates with what and how). Additionally, the benchmark has to be repeatable without much effort because it is foreseeable that most of the test-case generator will have non-deterministic behavior. This requires at least a few runs to get an impression about the variation of the generated output.

Technologies come with their specific constraints and behavior. For example, evolutionary algorithms (often used in Search-based Software Testing (SBST) (McMinn and 2011; Harman et al. 2015) need time to find a good solution. Depending on the problem, the time can take hours or longer. This behavior also decuples the input point in time from the test-engineer to the point in time the engineer can do follow up tasks (i.e., what other task can they do while waiting for the output of the evolutionary process?). If this is an important aspect, the benchmark suite has to be extended with an attribute measuring this discontinuity in the workflow. Making explicit lead time and working time can be a solution.

## Tanius: test case generation service

The competence center Test & Quality Assurance (TQA) of the Group IT supports and facilitates projects within the Volkswagen AG to realize adequate Quality Assurance (QA) and testing. Its Quality innovation NETwork (QiNET) (Poth and Heimann 2018) identifies, evaluates and develops quality related innovations. As innovations have to find their place in the market (Baregheh et al. 2009), the QiNET also focus on the potential deliverables with its research activities and cooperations. For a rigorous delivery, with focus on innovation, an innovative procedure was developed and established (Poth and Heimann 2023). As a vehicle to deliver the test-case generation as a service, the Test-Runtime execution (T-Rex) (Poth et al. 2022) is regarded as potential base. T-Rex can offer specific test-case generation options—called engines—without a large footprint. Each engine is an additional API endpoint with a micro-service to handle the specific engine configuration and scaling. On-demand T-Rex can commission resources on the cloud environment to handle test-case generation workloads with its elasticity. This is close to the generic model (recall Sect. 3.1), with zero static operation costs. The easy integration of new engines enables the possibility to add and evaluate, e.g., different AI approaches, and offer them parallel to users, as long they are complementing the test case-generation service offer. A strategic objective at TQA is to establish, besides T-Rex, the new Tanius (Test-Automation: Neural Intelligence Unlocks Scripting) capability for test-case generation for system tests, in particular end-to-end tests. The test-case generation journey started early in 2022, after analyzing the options proposed by the literature and by the evaluation of some open-source tools.

The QiNET decides to focus primary on the data centric and less on the model centric usage of AI technology. However, it needs sufficient comprehension about the models to manage the data centric approach efficiently. The QiNET does not see a strategic objective in the generation and maintenance of complex models. The area of customizing or developing new AI algorithms directly at TQA is out of scope. However, collaborations with academics to provide feedback on open-source research projects is viable.

**Fig. 5:**
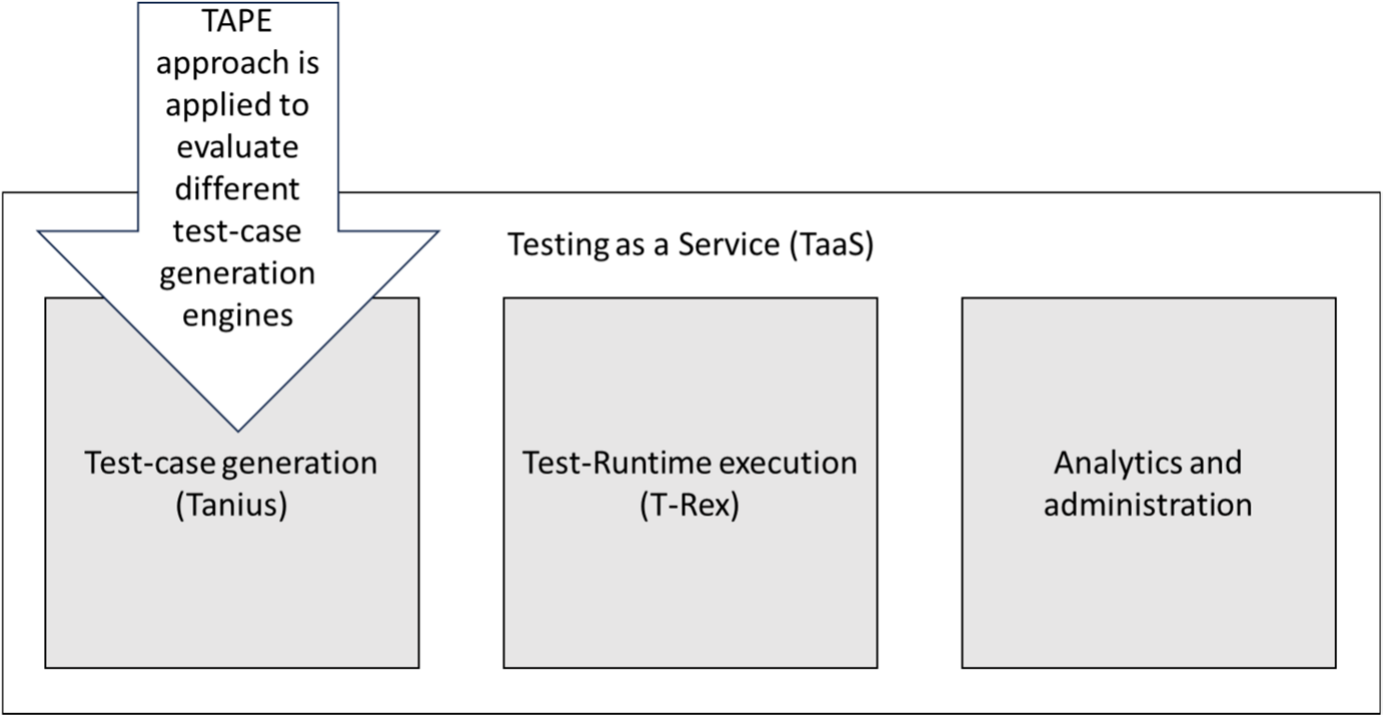
TAPE is applied to evaluate different test-case generation approches (engines) in TaaS context

On the one hand, TAPE is the evaluation approach. The generic approach is instantiated with test-case generation specific use cases for the benchmarking. On the other hand, Tanius (as part of TaaS like T-Rex) is the "IT system" which is evaluated, including the different integrated test-case generators. Tanius is the SUT from the TAPE benchmarks perspective (Fig. 5).

The specific domain knowledge about testing of the Group IT applications and service is the strategic asset which has been evolved and used. The usage also includes building services which are using and adapting the models. This requires a sufficient understanding of the models to make sustainable decisions.

### Constraints and requirements

Tanius is an expert system for test engineers to generate test-cases. The engineers are facilitated by their task to design and implement test-cases for a test automation of a software product or service.

Selected constraints for Tanius are:

- Fairness for both parties: the “data donator” (e.g., input data, training data) and the “consumer”, respectively the user (e.g., test engineers and the project) can be handled in the system(-design) adequately.
- No full job substitution by a machine is the objective. The test engineers are facilitated but still keep the responsibility for the task.
- Intellectual Property (IP) is respected by, e.g., using own or open data for training.
- Based on open-source.

Selected requirements for Tanius are:

- No data transfer to public internet (ChatGPT issues 2023) (maintain company IP (ChatGPT data use 2024)), i.e., service can run “airgap”.
- Data centric strategy; data centric over model centric implementation.
- No high budgets; apply a Frugal Innovation approach (Poth and Heimann 2018).
- Expandable; start with a valuable scenario and a simple technical setup and grow in complexity while learning.
- Modularized: different approaches can exist in parallel and be substituted with ease by better ones in the future.
- DevOps compatible; iterations are potential production-ready.
- Confidentiality; distinguish different data types such as public, internal (company), and confidential (project).
- Sustainable; deployed resources are in an adequate relation to resulted value unit.
- Cloud agnostic to run in private and public clouds.
- Integration with existing tool chain such as T-Rex.

Some requirements are interacting—examples are data centric, expandable and sustainable. It could be for example an expansion strategy to start with a model out-of-the-box, then over increments more and more embeddings are added via a chain or agent(s). The complex expansion chain allocates more resources than one optimized solution. Also, it can be faced a trade-off point between complex data handling for each request during inferencing and a fine-tuning training or transfer learning on the model level in advanced.

### Application

Based on these constraints and requirements, the TAPE approach is instantiated for Tanius. In the following, some examples are given on how this instantiation is realized. As a scenario the test-case generation for REST APIs is selected. This is motivated by the fact that many applications and services are offering APIs. Furthermore, REST API specifications are semi-formal defined, for example with the OpenAPI specification, i.e., schemas (OpenAPI 2024).

**Fig. 6:**
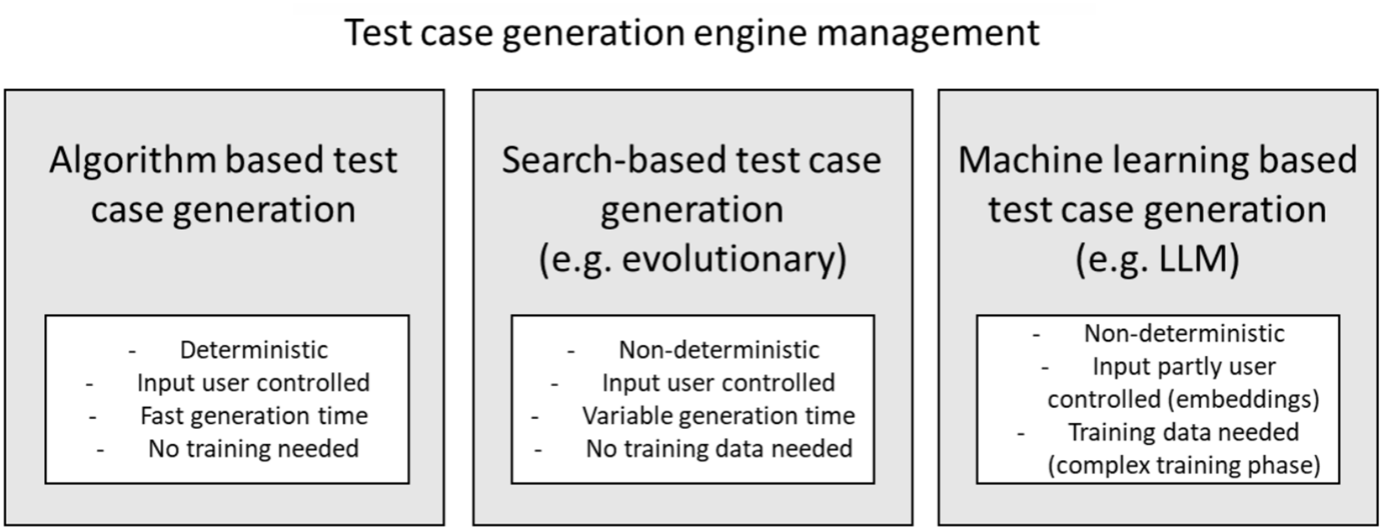
Handling of different test-case generation approaches as independent engines with the Tanius service

The generic structure of engine of Tanius is presented in Fig. 6. Different test generators could be used and integrated in Tanius. At the time of writing, three are in evaluation: *OpenAPI Generator* (Generator 2023), *EvoMaster* (EvoMaster 2024) and *StarCoder* (StarCoder 2024) (in particular, StarCoderChat). These represent different types of approaches, namely *deterministic*, *evolutionary* and *LLM*, as discussed in more detail next. Note that there are more tools available in the academic literature (Golmohammadi et al. 2022), and there has been scientific empirical work to compare them (Zhang and Arcuri 2023). Adding a tool to Tanius’s cloud solution takes effort, and can only be justified if it provides value to Volkswagen AG. As we will discuss in more detail next, a tool needs to provide upfront potential value before making the decision of spending resources to integrate it. For example, if it does not generate tests in a usable format such as JUnit, it would be of little to no value for our industrial needs.

Each of the three test-case generation approaches/technologies is handled as an independent engine (i.e., addressing the modularization requirements). This enables for each engine individual improvement and scaling based on-demand. The demand grows with its user acceptance and relative performance to other offered engines. So, mature approaches can be served to customers with the needed elasticity and feature rollout-speed. Each engine has its own life-cycle and release cycle (i.e., addressing the expandable requirements). This offers focusing on promising options, as the technologies for the engines can evolve with different speed. Furthermore, the modularized architecture offers adding a new engine, e.g., a new LLM, for evaluation without offering it as General Available (GA) to the users. Additionally, the isolation of the test-case generation approaches into independent services offered by the engines minimizes side effects. For example, this avoids that by accident the deterministic test-case generation approach becomes a non-deterministic feature from another approach by a mis-configured feature toggle.

In recent years, in the scientific literature and open-source community there have been proposed few approaches and tools for test generation for REST APIs (Golmohammadi et al. 2022). However, there is a huge difference between a proof-of-concept academic prototype (mainly developed to publish academic articles) and something that can actually be used at scale in industry. Based on industrial needs, the choice of what to integrate into the cloud infrastructure of Tanius was not driven by scientific research (e.g., to compare different algorithmic approaches), but rather to provide concrete value to the QA specialists at Volkswagen AG, in the long term. As such, in these decisions the ‘*human aspects*’ of how tools can be used and integrated plays a more important role than quantitative metrics such as the achievable code coverage of a tool. Algorithmic novelty has little to no value here, as what matters is the final benefits for practitioners in industry.

For example, considering the Tanius’s requirements listed in Sect. 4, “*integration with existing tool chain*” is a critical aspect of choosing a tool to integrate. In this particular case, the used T-Rex engine for running test cases is based on JMeter (Apache JMeter 2024). To be able to be used in Tanius, a test generator must provide an output format that is usable by JMeter, like for example JUnit test cases written in Java. It does not matter how good a tool is if then its outputs cannot be used. Mature, industry-ready tools might provide outputs in different formats, to cover different industrial needs, whereas proof-of-concept tools would likely just use one type of output.

Integrating a test generator into the cloud infrastructure of Tanius takes some time. Therefore, there are *usability* and *availability* aspects that need to be taken into account when making such a decision. Lack of documentation and usage examples can become a major showstopper. Furthermore, a tool should be under current development and maintenance, and not being a legacy, dead project. This is in case, for example, if bugs in it need to be reported, or for feature requests (like for example in the first interaction with the authors of EvoMaster^1^). Active open-source projects with timely engagement with the practitioner community in industry are necessary.

Before integrating these test generators into Tanius, we would not know how they would fare. Therefore, we tried to select tools based on the aforementioned requirements. We aimed to find tools using different kinds of technologies (e.g., evolutionary and LLM based ones), to be able to compare them, and so to identify their strengths and weaknesses when applied to the specific cases at Volkswagen AG, which is reflected in the TAPE benchmark suite.

#### Deterministic test-case generation

The static (i.e., does not need a running SUT) test-case generation is based on the OpenAPI Generator (Generator 2023). This is a set of utilities for developing, documenting and interacting with REST APIs. It is a very popular open-source project (more than 20 000 GitHub stars, at the time of writing), with nearly 3000 contributors, under active development since 2011. OpenAPI Generator was the first engine Tanius integrated into the T-Rex infrastructure. An upgrade of the OpenAPI Generator from version 6.0.1 to 7.0.1 (after more than one year) did not improve its performance. This indicates that the tooling did not improve regarding the relevant aspects related to the benchmark. Also, outside the generator no further performance relevant improvements were identified. This engine is still in State 1 of Tanius’s integration, and no significant improvement concepts are identified to initiate a State 2 development. However, the life-cycle with for example release-updates is established.

#### Evolutionary test-case generation

EvoMaster (EvoMaster 2024) is an academic open-source tool, based on evolutionary computation, under development since 2016. It is a mature tool under active development, with existing funding for the FOSS project for the next years from research grants. With the strategic decision not to develop AI algorithms, the Free & Open Source Software (FOSS) license is acceptable. However, the license is limiting in the case that a small code change would give improvements (and the code should not be shared to protect company IP which requires the “code-adaption”). T-Rex faced this case once in the past and was able to deliver a large added value to projects by changing a few lines of code to call a specific proprietary library in an Apache 2.0 licensed component. EvoMaster v1.6.1 is used in a basic setup to generate system tests based on the OpenAPI specification for the SUT in State 1. At the time of the experiments, EvoMaster did not exploit any additional information given by the OpenAPI specification, such as *example* or *default* entries. This information would be useful to generate at least one positive test case without heavy computation, or in some cases would help to overcome a plateau of the evolutionary approach. The State 1 implementation shows that available data is not used but would lead to significantly better results, i.e., better benchmark performance. To “guide” or at least let the evolutionary algorithm start in the right direction, promising extensions are currently investigated by EvoMaster’s authors based on the feedback from this work.

#### LLM-based test-case generation

**Fig. 7:**
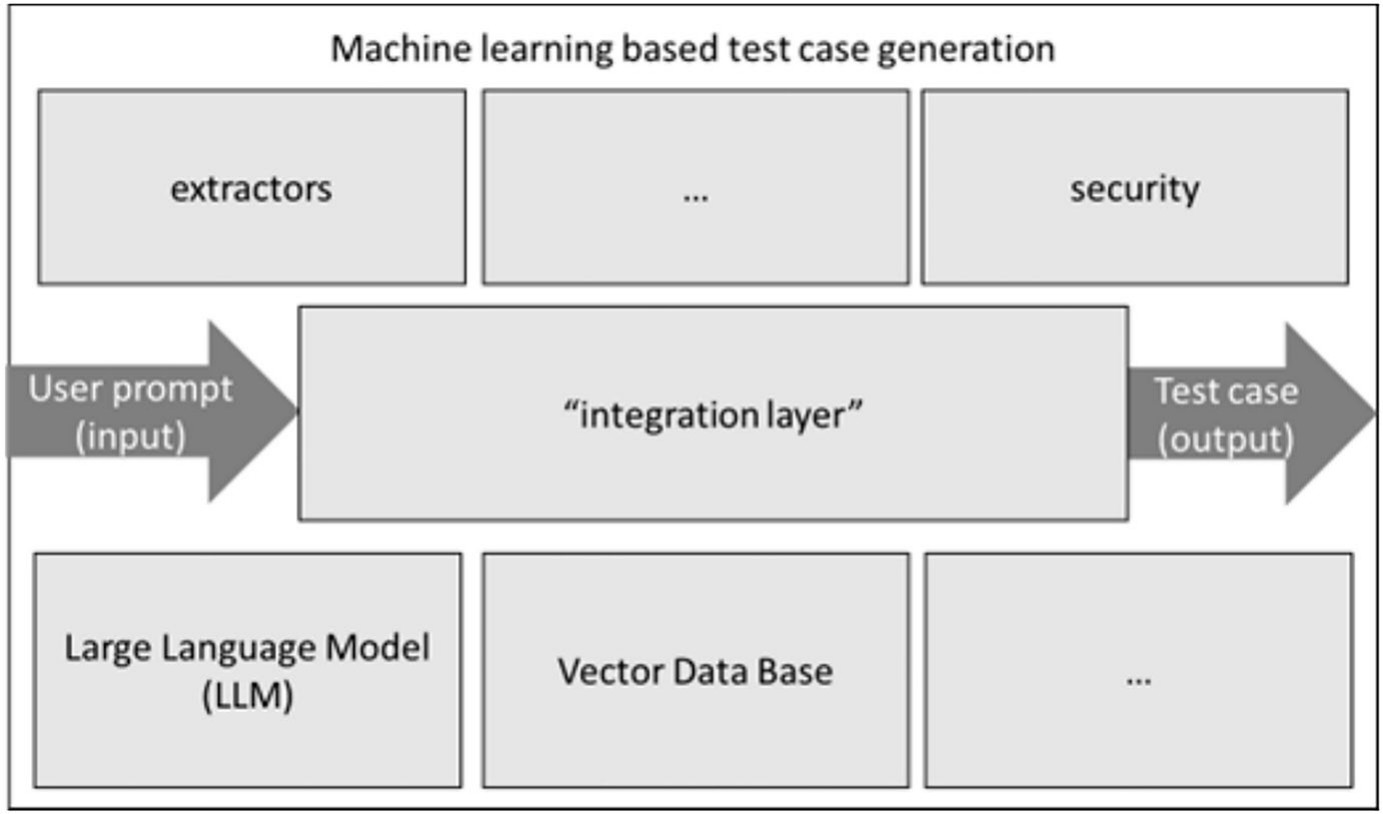
Generic target architecture to build a sophisticated service with incremental releases

The first available LLM for the evaluation we found, fitting the constraints about fairness and open-source, was StarCoder (StarCoder 2024). Currently there are many LLM solutions out there. We chose StarCoder because it was among the most popular LLM tools for code tasks, and its description and documentation did fit our needs. However, without supporting empirical evidence, we cannot say for sure if other LLM-based tools could had been better. Furthermore, integration into LangChain (LangChain 2024) is available. This is the base for an incremental development via releases delivering new features and capabilities to evaluate the potentials of the technology. Figure 7 shows a generic architecture using, for example, LangChain as integration layer to add features to improve the performance in the benchmark. The vector database stores the domain respectively company specific embeddings. The three dots indicate that other aspects can be addressed (in the future), too. The LLM is used with the prompt from the user without additional and advanced prompt engineering methods. In most cases (non-determinism) the given information is used to generate useful test-cases. However, not all test-cases are executable without at least some small syntactic, manual adjustments. This type of problem has been addressed in the scientific literature, with techniques such as “self-reflection” (Madaan et al. 2024). However, those did not seem to be available yet in “usable” tools at the time of our experiments in this paper. Furthermore, the resources allocated by the deployment are significantly larger than for the other engines. Currently, the investigation around different prompt engineering approaches is evaluated, such as templating or examples selectors to generate more sophisticated tests. Oriented on Fig. 7, next iterations will be implemented.

**Fig. 8:**
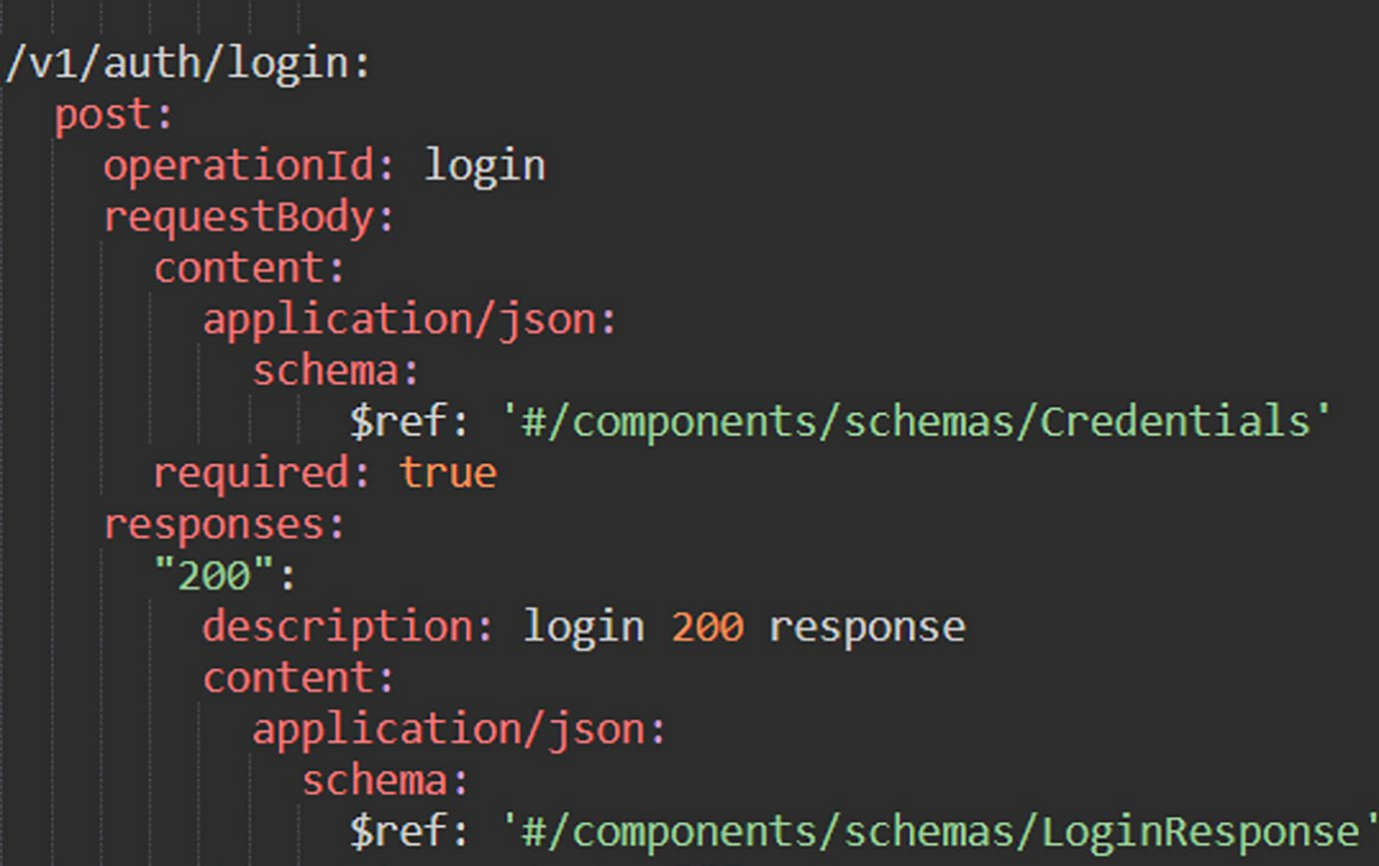
OpenApi specification which describes a login endpoint

Figure 8 shows an excerpt of an OpenAPI specification for an endpoint in one of the API used in our case study in the next section. Table 1 shows tests generated by each of these three tools described in this section.

**Table 1:** Example of generated tests for login endpoint

Tool	Generated Test/s	Description
OpenApi Generator (OAG)		Only one test was generated. The adjustments for the test were implemented so that the login was successful. The test called the real target object, when it was created/generated
EvoMaster (EM)		Several tests were generated. In the examples presents a successful (positive) and unsuccessful (negative) test. The tests called the real target object, when they were generated/created. This means that the response was sent by the target object and no hallucinations were created
StarCoder (SC)		Also with Starcoder several tests were created, and in the examples they can be seen a positive as well as a negative test. The tests were not carried out against a real test object, when they were created. In the second test, for example, can be seen an "error_description" in the body, which is not sent in this form from the target object. There is no error_description property in the response of the backend system. The same is the case with the message "Bad credentials". This is a hallucination from the LLM model. This need to be fixed before running against the real target object

## Evaluation

In this industry report, we aim at answering the following research question (RQ): *what practical benefits can TAPE bring to an enterprise such as Volkswagen AG?*

TAPE, as a technology evaluation approach, is used to evaluate, respectively benchmark, different technologies. For the evaluation of TAPE, an example scenario for test-case specifications, respectively test-case generation, is chosen. The test-case generators use different technologies and are evaluated for (a potential) integration into Tanius. The scope is to derive from interface specifications (i.e., an OpenAPI schema) the test cases to validate the implemented API against its specification. As pre-condition for the benchmark, it is assumed that the OpenAPI specification with example test-data exists and the corresponding SUT is prepared (e.g., up and running). Furthermore, adequate roles and rights for execution are available, e.g., current authentication info is available.

For the empirical evaluation, an auth-service and user-service developed at Volkswagen AG are selected, which satisfy these pre-conditions. Additionally, as it is a snapshot of real production code, it is a representative case to give trust in the results which can be realized in other real-world products and services, too. The motivation to select such services is that practically all SUTs have to deal with this topic, i.e., they need to use these services for their operation (e.g., to check if the provided input credentials are valid). The attribute for the auth-service is different auth-approaches, which are defined in the cases *JWT* (JSON Web Tokens) and *Key* (static secret identifier, used like a combined userid/password). The cases represent specific technologies such as based on keys or tokens. In practice, the same auth-service API was tested in two different scenarios, considering the testing of two different subsets of HTTP endpoints declared in its OpenAPI schema. The auth-service has a simpler functionality and less complex API interaction as the user-service. Table 2 shows some properties of these two APIs, including lines of code for their business logic, and the number of HTTP endpoints.

**Table 2:** Meta-data about the services

Service	Lines of Code	Endpoints
Auth-service	5750	3
User-service	8591	21

The auth-service is simpler than the user-service. In the TAPE approach, the auth-service is like a T-shirt size S and user-service a M. This also implies that the test generators need more advanced techniques to generate useful test-cases for the user-service.

The detailed definition of the cases is realized by a 100% test suite used as a “reference”. In other words, for the sake of the experiments for this case study, for these APIs an existing, curated test suite is used as optimal, ideal reference solution. This is an actual test suite manually prepared by engineers at Volkswagen AG as reference, before running our experiments. As such, it is not necessarily a theoretical optimal test suite for the chosen SUT. With this 100% definition, it is still possible that an automatically generated test suite in the future outperforms the reference defined by the engineers. The 100% values are based on the time an engineer needs to build the reference test-suite. The time can be multiplied by the hour rate to get a monetary value—which is not needed for evaluation in this paper. In this evaluation, only time is measured. To avoid possible bias in the comparison between the percentage values between the different tools, the same engineers evaluated all the results.

Table 3 presents our results of the TAPE benchmark analysis on the auth-service and user-service, using four different approaches: test suite reference manually written by engineers (**MAN**), generated by OpenApi Generator (**OAG**), EvoMaster (**EM**) and StarCoder (**SC**). As EM and SC have non-deterministic behavior, the test-generation was repeated 3 times. The State 1 uses versions of the tools from 2023, whereas State 2 from summer 2024. In all states the tools are used where possible in their out-of-the-box configuration. Only a few parameters and attributes are set to run the benchmark (e.g., to set for how long to run them). With sophisticated tuning additional potential could be levered. Especially for the LLM a high potential is expected, but the effort to exploit this is also high. The results are presented in detail level (recall Fig. 3) based on TAPE. Four different metrics are used:

- Input (I): engineer effort to prepare the data for the test generation such as ensure that the file-format fits to the tool, add data in tool accepted format, for examples to represent acceptance criteria
- Verification & Validation (V): engineering effort for verification & validation of the generated tests-cases about correctness and completeness, to identify gaps such as is the test executable, is the test-code “readable” and is the test traceable to the examples
- Gap (G): engineering effort to close the identified gap by fixing incorrect tests, add tests to reach at least the baseline of the MAN test-suite
- Optimization (O): it represents the gained effort performance against the initial 100% reference through the facilitation by the test-case generation.

**Table 3:** Benchmark results for two value units and their test cases for Manual (MAN), OpenAPI-Generator (OAG), EvoMaster (EM) and StarCoder (SC)

State 0	Auth-Service: Key	Auth- Service: JWT	User-Service
MAN	100%	100%	100%
State 1	Auth-Service: Key	Auth-Service: JWT	User-Service
OAG	I: 1.25%	I: 0.91%	I: 35.7%
	V: 3.13%	V: 4.55%	V: 13.3%
	G: 65.63%	G: 54.55%	G: 41%
	O: 29.99%	O: 39.99%	O: 10%
EM	I: 1.25%	I: 0.91%	I: N/A**
	V: 6.25%	V: 5.45%	V: N/A**
	G: 45%	G: 38.64%	G: N/A**
	O: 47.5%	O: 55%	O: N/A**
SC	I: 1.25%	I: 0.91%	I: N/A
	V: 6.25%	V: 5.45%	V: N/A
	G: 34.38%	G: 29.55%	G: N/A
	O: 58.12%	O: 64.09%	O: N/A
State 2	Auth-Service: Key	Auth-Service: JWT	User-Service
OAG	I: 1,25%	I: 0.91%	I: 35.7%
	V: 3.13%	V: 4.55%	V: 13.3%
	G: 65.63%	G: 54.55%	G: 41%
	O: 29.99%	O: 39.99%	O: 10%
EM	I:1.25%	I:0.91%	I: 10.5%
	V:6.25%	V: 6.25%	V: 39%
	G:46%	G: 40.64%	G: 20.5%
	O:46.5%	O: 52.2%	O: 30%

These four metrics were manually calculated based on the time needed by the engineer effort for each of the 4 tasks in relation to the 100% reference baseline. As the reference baseline was measured in time the optimization also is measured in time.

As we can see in Table 3, between State 1 and 2 no improvements on the OAG can be seen, because the releases do not deliver new features to enhance the results. EM can enhance its results in State 2 by running successfully against the user-service. In State 1 EM was not able to handle the API complexity respectively semantic on the test-data level which is marked with N/A** in the table. The user-service was not benchmarked with SC (N/A in the table), as that (old) model was no longer available when we ran the experiments. For the same reason, there is no SC in State 2, because the new version did not run out-of-the-box.

Table 3 shows that no code-generation engine of Tanius outperforms the quality of handcrafted reference test cases implemented manually by the engineers. With the growing complexity of the test-object from the auth-service to the user-service the realized facilitation respectively optimization (O) by the tools drops. However, the LLM approach with StarCoder (SC), especially StarCoder chat, looks promising. Especially, this is the case with the many improvement options LLMs are offering and not used in the current state. In the next state extensions, oriented on the approach presented in Fig. 6, further performance improvements are expected, as current investigations are showing. Also, an interaction of EvoMaster (EM) and SC can be an interesting option to overcome the limitations of the two different approaches. Overall, the TAPE approach represents a technology management tool to evaluate the performance of current states and helps to derive promising strategic options for Tanius.

Regarding variability in the results, for EM no significant output variances were observed. For SC, several requests were made to expand the test suite (token limit) and the results were partly better and the test suite was filled with new tests. Recall that OAG is deterministic, so there is no variance in its outputs.

Regarding the readability of the generated tests, for SC the engineers at Volkswagen AG considered its generated code as easy to read and easy to expand. The naming of the functions and the creation of the test data by the SC was acceptable. On the other hand, the name of tests generated by EM are all in the form *test01()*, *test02()*,…, *testN()*, which is not user-friendly. For OAG, the code itself was readable and all libraries, packaged, models etc. that were needed to run the tests were also generated. To further assess the quality of the generated tests, the JVM of the SUT was instrumentalized with JaCoCo (JaCoCo 2024) to measure the C0 and C1 coverage. C0 measures the coverage of statements, whereas C1 measures the branch coverage of the source code. Note that computing these metrics was done only for the sake of this empirical evaluation. Generally, for black-box testing, no code coverage is measured, as tests are designed based on the requirements of the SUT. Also, keep in mind that it is not expected that system tests have the same high code coverage as unit tests. Detailed functional tests are on the unit test level. A professional engineer will focus on system level tests development only on the related system level aspects. This avoids wasting resources in double testing and reduces the maintenance overhead in the long-term.

The test-generation tools do not have this “higher semantic”-knowledge to keep focus on system level aspects. This can lead to too many tests (derived by an automatism because it is possible to derive all these tests). An engineer also has to delete “senseless” tests generated by the test-case generator (as part of Verification &Validation). At least EM tries to combine test-aspects to a minimal set of tests which is the outcome test-suite. This example shows that it is not easy to define by simple metrics what a holistic “test quality” is.

Table 4 presents the results for the executed tests. Note that these test suites are the combination of the ones generated for the Key and JWT subsets of functionalities (i.e., subsets of endpoints in the OpenAPI schema). By design, tests generated by EM are executable, as they are syntactically valid. The tests generated by OAG and SC in most cases need some “adjustments” to be executable. Fixing those generated tests required manual effort, but it was manageable. In Table 4 the adjusted versions are measured. Adjusted tests are marked with * in Table 4. The manual reference test suite (MAN) was also evaluated to compute quality metrics such as C0 (statement) and C1 (branch) code coverage against the reference SUT for which the OpenAPI specification is defined (Table 4).

**Table 4:** The C0 and C1 coverage of the generated system tests (TC) measured on the JVM of the SUT (Auth- & User-Service)

State 0	Auth-Service	User-Service
MAN	TC: 16	TC: 56
	C0: 52%	C0: 42%
	C1: 27%	C1: 25%
State 1	Auth-Service	User-Service
OAG	TC: 3*	TC: 21*
	C0: 28%	C0: 28%
	C1: 11%	C1: 16%
EM	TC: 11	TC: N/A**
	C0: 30%	C0: N/A
	C1: 16%	C1: N/A
SC	TC: 12*	TC: N/A
	C0: 30%	C0: N/A
	C1: 15%	C1: N/A
State 2	Auth-Service	User-Service
OAG	TC: 3*	TC: 21*
	C0: 28%	C0: 28%
	C1: 11%	C1: 16%
EM	TC: 8	TC: 43
	C0: 29%	C0: 33%
	C1: 13%	C1:19%

**RQ**: *None of the automated test generators integrated in Tanius gave better results than the manually developed tests*, as shown in Table 4. Still, these tools can provide a valuable starting point for the test engineers, instead of writing manual tests from scratch. However, to be effective in such a task, the readability of the generated tests is paramount. Currently, the tool-facilitation does not generate enough tests to reach the quality of a handcrafted test suite. However, the risk exists that the quantity of generated tests become an issue—the engineers have to “filter” useful tests for the regression test-suite and drop other tests to reduce testing resources for execution, administration and maintenance of the test suite.

## Discussion

In the professional opinion of the test engineers involved in this study, the generated test-cases were in all tools at least a good starting point to build a regression test-suite. Some test-cases were easy to map to the corresponding baseline test-cases developed manually, but other test-cases needed more time for verification and validation, as shown in Table 2. Interesting is that, e.g., EM “explored” the SUT based on the given information and detected a concerning fault in the auth-service. As the auth-service in the benchmark suite is a snapshot of the real service, the fault was still there in production, and so was promptly fixed for the next production service release. This additional insight was an unexpected added value which is not directly visible in the presented metrics. This is because the gap was still existing to the baseline tests (i.e., the test suite used as reference for comparisons did not detect such a fault). Similar added value results occurred in the user-service were EM found also an “undefined” behavior which was fixed in the production code (but kept in the benchmark version of the code as “hidden issue” for performance measures). The additional bugs found by the tools would only be visible for the case that the entire generated test-suite is on the level of the manual engineered test-suite with values over 100%—currently all evaluated tools are far away from this quality level.

The discussion reflects the contribution of the benchmarking in terms of the TAPE approach defined metrics from Sect. 3:

1 $${\text{AddedValue}} = {\text{ValueUnit}}{-}{\text{ResourceDemand}}$$

2 $${\text{CostOfValueUnit}} = \# {\text{AddedValue}}/{\text{ReleaseCosts}}$$

From the Tanius user perspective: The positive impact on AddedValue is given by that testing ensures the full ValueUnit delivery and all 3 test-generators have no ResourceDemand in service, because only direct for the user value generation resources are needed during the test-case generation job (without significant static footprint in the evaluated implementation). During development, the tools allocate some resources, but as they can run as on-demand container workloads, their impact is negligible (however, the LLM needs significantly more resources than the other two approaches). The positive impact on CostOfValueUnit is given by the reduction of the ReleaseCosts—the test-generation facilitation significantly reduces the work to setup the test-suite (Table 3), which reduces the ReleaseCosts.

From the Tanius service provider perspective, the Optimization (O) realized (Table 3) is the ValueUnit which is to be sold to the Tanius’s users. Each user that requests to generate the tests allocates infrastructure resources for computation (ResourceDemand). For OAG and EM this is small (spin up a container with normal CPUs on demand for a few minutes), but for SC it is not so simple to spin up a container. This is because LLMs needs a significant amount of time to start, warm-up, etc., and requires specific hardware such as GPUs—there comes with much higher resource allocations and costs for overheads and processing. However, in most cases, the costs for an engineer’s working hour are much more than additional costs for the LLM, so it is still an AddedValue for the Tanius customers. The ReleaseCosts to build the features of Tanius have an impact to the CostOfValueUnit. Complex adoptions such as for LLMs drive up the ReleaseCosts. This becomes an issue if the usage to refinance the costs is not growing significantly, too. This shows that the Tanius team has to look what efforts are spent into a new feature release for SC improvements, by, e.g., adding states and complexity into the chains for a higher Optimization score.

Based on the applied benchmarking, it looks like that EM is a good trade-off between AddedValue, respectively Optimization, and ResourceDemand (compute, elasticity, etc.) and ReleaseCosts (complexity efforts, etc.). However, LLMs such as SC have a high potential for further Optimizations by adding techniques into the chains and agents—coming with a significant effort for the integration and only a high adoption with frequent usage (#AddedValue) justifies the heavy lifting of the LLM chains and agents. However, LLMs evolve fast so that also out-of-the-box usage of newer model release can improve Optimization without many tuning and adoption efforts.

Furthermore, all benchmarked tools and technologies are only a facilitation for the test engineers—no approach generates test-suites on the quality of a professional test engineer. With the test-object complexity the generated test-suite quality drops which leads to more work for the engineers and less overall optimization of the efforts and time. However, the tools can help in most cases to build an initial test suite in a short time which is a good base for the engineers to iteratively and incrementally enhance the test suite.

Another important aspect to consider is “test maintenance”. With the evolution of an API, existing written test suites need to be updated as well. This requires time and effort. AI facilitation would be useful here. However, at the current time, no user-friendly tool seems to exist addressing this problem.

A potential ethical risk of the proposed approach (including the case study) is that the current objective to facilitate test engineers in their daily routine work of software test-case design and implementation can take a turn into a job-killer. Currently, the IT market lacks skilled workforce and every facilitation is welcome. But, this can change in the future. Then, an over increments and iterations of the versions/releases and their improvements optimized test-case generation can become a job-killer. Especially junior engineers carrying out “non-expert” tasks will face an issue. A new generation of test engineers could be kicked out of the job before they can accumulate enough experience to do the job of a senior.

## Threats to validity

### Internal validity

The threat to internal validity mainly lies in the instantiation of the benchmark. The risk is that the value units and the attribution and the derived cases do not fit well. Then, the performance of the implementation journey does not optimize value units as expected. To reduce this kind of threat, all authors checked the value units and the corresponding benchmark suite.

Internal validity concerns whether our conclusions may be wrong due to methodological errors. A possible issue is that we used some wrong or incomplete assumptions for the TAPE approach. We aimed at mitigating this issue by iterative refinement of the models to keep the changes in each step small. An overall threat is to focus on the wrong scenario and therefore optimize the wrong thing. This is mitigated by continuous market observation to detect disruptive approaches early.

### External validity

The threat to external validity mainly lies in the limited evaluation setup used in our study. We assessed the evaluation setup and environment to fit the objectives of this work. But, we could not mitigate this threat in the current evaluation setup, and we aim to collect further data from other evaluation setups as future work.

Furthermore, the work was only conducted within Volkswagen AG and does not address other companies and their cultures. We cannot mitigate this as long as we stay in this one multi-national enterprise. However, the evaluation setup is a multi-cultural team based on different countries.

The straightforward refinement proposed in the TAPE approach may not work in all situations and scenarios. Future versions of the TAPE approach should have mechanisms to detect if it is operated in a context which is not “compatible” with the TAPE approach.

The benchmarks may not be so easy to define and be biased. Currently, this is only mitigated by the checks of the authors. To mitigate this issue systematically, future versions of the TAPE approach should have mechanisms to assess the benchmark suites about “quality”.

Related to the case study with the LLM evaluation: Fair Intellectual Property (IP) handling is an open issue in the current state of using AI, especially in the context of foundation models such as LLMs. We tried to handle this by selecting a LLM which is FOSS and was trained and validated with FOSS code as data. However, some aspects are still unclear, such as what licenses get the test-cases with a more or less large part of generated code. At which point of changed generated code by a human engineering triggers the proprietary right attributes of an outcome to the human? Is there a relation between the license proportions of the training data and the generated code? For example, would training data (i.e., code in this case) released under GPL imply that all outcomes have to be GPL too? Or is a LLM more like a software engineer who saw a lot of code, including a lot of GPL code, as inspiration of own creations for which the engineers can choose a license? This kind of risk is mitigated by the high modularization and ease to change components, which ensures to stay compliant with upcoming regulations such the EU AI Act (EU 2024) and its state-of-the-art interpretations by courts.

### Construct validity

The threat to construct validity mainly lies in the randomness in our study. To reduce this kind of threat, we select an approach with the option of a long-term benchmarking with more optimization releases per technology. This enables us to see the performance progress per release for each technology. It is possible to define a starting point and benchmark upcoming release to keep up-to-date or have a look on past releases to see the trend. The second approach can help to focus in a “good running trend” of a technology and the first approach to see if the trend is still there and justifies the focus with investments, etc.

A conceptual issue is a lack of transparency which has to be established by design. This is motivated that the benchmark cases in detail have to be kept away from broad public, to avoid that they become part of training data for LLMs. One example is if the benchmark cases are published on GitHub, then StarCoder will be trained on it. If the benchmark cases are used for training the benchmark, results can become invalid as the LLM knows the “perfect” answer from the training. Furthermore, there is a risk that people try to manipulate the benchmark results. One possible example could be reverse prompting. Reverse prompting uses the “perfect” answer as input for a prompt and asks the LLM for the corresponding prompt to produce the “perfect” answer.

The adoption and tailoring of existing measurements were applied when possible. The development of specific proprietary and private measurements is based on state-of-the-art approaches from the literature in Sect. 8. Our analysis confirmed that our constructions are internally consistent and scoring well.

One potential issue is that we do not have a long-term performance of the proposed approach. A good base would be a study about different products and services over their life-cycle to evaluate the performance also with other measures in a post-mortem. This evaluation about the overall life-cycle performance of the proposed approach within different product and service environments is a topic for future work.

Another potential issue is that, depending on company or regional culture, the derivation of key elements of the proposed approach such as the benchmark cannot be objective, e.g., with an uncommunicated “implicit objective” such as to keep people in work and avoid workforce reduction the benchmark with the scenario and cases can be designed to support the objective. A biased or not sufficient representative benchmark for a scenario can lead to wrong indications and decisions. Our recommendation is to define at least all elements of the proposed approach in mixed teams to avoid this kind of design failures.

## Related work

### Benchmarking

In the context of AI, the Turing Test is an often referenced example (Copeland 2000). However, the pass/fail decision is not enough to see relative progress in the evolvement of a system. In Hernandez-Orallo (2000), it is proposed as relevant aspects such as non-Boolean decisions, factorial to evaluate specific domains, and meaningful within a context to evaluate the performance. A way to evaluate performance is benchmarking. A good benchmark handles according to Poth and Heimann (2018) relevance, reproducibility, fairness, verifiability, and usability. In Huppler (2009), aspects such as economical and limitations come in, which have a high impact in practitioner setups. For practical relevance, it is also important to have the experiments in mind in Hothorn et al. (2005). For code generation, existing different benchmarks such as CodeContest (Li et al. 2022), APPS (Hendrycks et al. 2021), and HumanEval (Chen et al. 2021) exist. CodeContest contains 165 code problems and was used to train AlphaCode and other models. APPS is for Python, and includes for the training and test set together 10,000 problems. HumanEval is based on 164 hand-written problems. In Mazumder et al. (2022), a data-centric benchmarking approach for AI models is proposed.

### Search-based software testing (SBST)

In Harman et al. (2015) and (Poth and Heimann 2018) many years of research outcomes in the area of SBST are presented. In Golmohammadi et al. (2022), a survey about tools for REST API testing is presented. In Zhang et al. (2022), the tool EvoMaster is evaluated in an industry context for micro-service testing. The tool can be used in black- and white-box testing setups (Arcuri 2020). To compare the state-of-the-art in SBST, annual tool challenges exist (Biagiola et al. 2023; Gambi et al. 2022). The SBST eco-system has been established throughout the years with an active community, with continuous new developments. This makes it possible to choose a tool for the specific needs.

### LLM for code generation

Twelve different LLMs, including ChatGPT and StarCoder, are evaluated in Liu et al. (2023) about their performance to generate test-cases with the HumanEval benchmark. In Ouyang et al. (2023), ChatGPT (GPT 3.5 and GPT 4) was asked five times to generate code for selected benchmark suites. The results were compared, and high non-deterministic outcomes were observed with a significant semantic and syntax difference of the code outcomes. (Tang et al. 2023) compared a LLM (ChatGPT) with a SBST tool (EvoSuite). An interesting approach is presented in Lemieux et al. (2023) which combines SBST with a LLM based approach, to overcome plateaus of the evolutionary algorithms. To improve the code-generation performance with focus on exception handling facilitated by LLMs, an approach called KPC is proposed in Ren et al. (2023). The approach can be reduced to iterate with prompt refinement based on the assessment of the outcome, as long as it does not fit the demand.

### Robotic process automation (RPM)

RPM is not a direct scope of this work, but it offers some inspiration with the Total Value of Ownership (TVO) approach, which is proposed in Willcocks et al. (2018) by having the Total Benefits of Ownership (TBO) greater than the Total Cost of Ownership (TCO).

## Conclusion

The proposed TAPE approach was developed to handle new technology adoptions, by focusing on realized added-value benchmarking over the delivered releases. As it is difficult to define a-priori detailed requirements about technology (which can evolve fast), adopting a generic approach was established to refine the requirements via benchmark scenarios. The benchmark suite can evolve with the technology and the learnings from stakeholder feedback. With a multi-level refinement, from an abstract theoretical model (i.e., a generic model) to a specific technology adoption case, TAPE can be used at an early phase of product development or service delivery. However, the refined case specific benchmarks can be adjusted to new requirements and demands or learnings during the life-cycle of the product or service. In the case that different technologies are used to address the same case, respectively are measured with the same benchmark, it can show the relative performance of each (technology) release. It can be used as maturity indication because often mature technologies offer smaller progress steps than emerging technologies. Especially for the many different AI technologies, this can be useful as long as not all are compatible or combinable to get an overall performance improvement.

The proposed TAPE approach is a promising procedure to address the pattern “*new technology adoption for value generation *via* automation*”, which is not limited to the presented Tanius case study context. Application of the proposed generic systematic approach to facilitate automation in other technologies and projects is on the backlog. For the specific Tanius case study, TAPE confirms the strategy with automated test-case generation as facilitation for the engineers to improve the overall testing performance. Furthermore, the TAPE approach indicates for a short-term focus EvoMaster, because EvoMaster seems “mature”, can be integrated into workflows and delivers useful test-cases. The long-term scope can move more to LLM-based approaches, but currently the integration and adoption is still more difficult and cost intensive.

After five years focused on scaled test-runtimes with T-Rex at Volkswagen AG, a next phase has started with focus on test-case generation facilitation for requirements validation with the Tanius service. Tanius integrates different technologies to a workflow which facilitates the test-engineer tasks around test-case specification and design. The goal is to help, but not replace, test engineers that need to validate REST APIs based on their requirements. The results of our case study show that emerging, existing AI techniques can already be of value for test engineers in industry, working in large enterprises. At the current state of the evaluated tools, we see that EvoMaster is a good starting point for AI-facilitation because in our usage it always generated executable test-case code. The code generation can be improved without advanced prompt engineering by only adding examples to the OpenAPI specifications of the REST API. Also, it is fast and has an adequate resource footprint for each request. LLM approaches come with a much bigger footprint and need much more tooling to realize similar results—however the long-term potential looks more promising. Unfortunately, legal and compliance topics are still open in the LLM context (Poth et al. 2024), which slows down adoption and general usage in many cases.

Next steps are to elaborate enhancements and extensions of LLM and evolutionary approaches for a better test engineer facilitation by designing and implementing test-cases. In particular, several areas of improvement were identified for EvoMaster, and are currently under investigation and implementation by its authors. However, the strategic focus is to evolve the data driven approach about the knowledge domain “testing Group IT applications and service”.

## Data Availability

The compared tools are all open source, and available online. The case study is industrial, and it is an intellectual propriety of Volkswagen AG. As such, it cannot be made available.

## Footnotes

1 https://github.com/WebFuzzing/EvoMaster/issues/820
